# Extraperitoneal lymph node dissection in locally advanced cervical cancer; the prognostic factors associated with survival

**DOI:** 10.4274/jtgga.2016.0202

**Published:** 2017-06-01

**Authors:** Mehmet Faruk Köse, Mine Kiseli, Günsu Kimyon, Reyhan Öcalan, Müfit Cemal Yenen, Gökhan Tulunay, Ahmet Taner Turan, Işın Üreyen, Nurettin Boran

**Affiliations:** 1 Department of Gynecologic Oncology, Etlik Zübeyde Hanım Women’s Health Training and Research Hospital, Ankara, Turkey; 2 Department of Gynecologic Oncology, Gülhane Training and Research Hospital, Ankara, Turkey

**Keywords:** Cervical cancer, extraperitoneal, Lymph node

## Abstract

**Objective:**

Surgical staging was recently recommended for the decision of treatment in locally advanced cervical cancer. We aimed to investigate clinical outcomes as well as factors associated with overall survival (OS) in patients with locally advanced cervical cancer who had undergone extraperitoneal lymph node dissection and were managed according to their lymph node status.

**Material and Methods:**

The medical records of 233 women with stage IIb-IVa cervical cancer who were clinically staged and underwent extraperitoneal lymph node dissection were retrospectively reviewed. Paraaortic lymph node status determined the appropriate radiotherapeutic treatment field. Surgery-related complications and clinical outcomes were evaluated.

**Results:**

The median age of the patients was 52 years (range, 26-88 years) and the median follow-up time was 28.4 months (range, 3-141 months). Thirty-one patients had laparoscopic extraperitoneal lymph node dissection and 202 patients underwent laparotomy. The number of paraaortic lymph nodes extracted was similar for both techniques. Sixty-two (27%) of the 233 patients had paraaortic lymph node metastases. The 3-year and 5-year OS rates were 55.1% and 46.5%, respectively. The stage of disease, number of metastatic paraaortic lymph nodes, tumor type, and paraaortic lymph node status were associated with OS. In multivariate Cox regression analyses, tumor type, stage, and presence of paraaortic lymph node metastases were the independent prognostic factors of OS.

**Conclusion:**

Paraaortic lymph node metastasis is the most important prognostic factor affecting survival. Surgery would give hints about the prognosis and treatment planning of the patient.

## INTRODUCTION

Cervical cancer is a major health problem worldwide and the most common cause of cancer-related death in women from developing countries. Survival and management of cervical cancer depends on the stage of the disease, which is determined by the principles of the International Federation of Gynecology and Obstetrics (FIGO) revised in 2009 (1). Five-year survival rates achieve 88-100% in stage Ia-b disease, whereas in advanced stage, it barely reaches 50%. Treatment of early-stage disease comprises mainly surgery, whereas higher stage disease is managed using chemoradiation. Although the staging system does not include lymph node involvement, radiation therapy principles are determined according to the extension of affected lymph nodes. Inaccuracy of clinical staging, which reaches 50-56%, makes pre-treatment nodal staging and future research about the topic more important (2).

With the additional finding of lymph node involvement as the most important prognostic factor for cervical cancer, assessment of lymphatic involvement has gained greater importance. Surgical evaluation of lymph nodes is a reliable method and may be performed either transperitoneally or extraperitoneally. Surgical staging of cervical cancer has been implemented commonly since the 1990s when the extraperitoneal technique was discovered because the transperitoneal approach was shown to increase postradiotherapy complications and thus morbidity such as urologic and gastrointestinal problems (3, 4). Extraperitoneal lymph node dissection with chemo-radiotherapy has been investigated heavily in patients with locally advanced cervical cancer, but in developing countries with low income, it brought about many difficulties in practice.

Surgical staging in order to evaluate paraaortic lymph node status was recommended in the 2015 National Comprehensive Cancer Network (NCCN) guidelines in patients with locally advanced (stage Ib2-IVa) cervical cancer (5). Treatment modalities differ in patients with positive paraaortic lymph nodes. The data of long-term outcomes are required because surgical staging is recommended and used more frequently. This retrospective study aimed to investigate clinical outcomes as well as factors associated with overall survival (OS) in patients with locally advanced cervical cancer who underwent extraperitoneal lymph node dissection and were managed according to their lymph node status.

## MATERIAL AND METHODS

In this retrospective study, 240 patients with locally advanced stage cervical cancer who had extraperitoneal lymph node dissection in the gynecologic oncology clinic from January 1998 to January 2013 were enrolled. Data about patient characteristics, treatment, histology, stage, and follow-up were collected from medical records. The study was approved by the local ethics committee (2016/216). All patients were clinically staged preoperatively in accordance with the FIGO staging system (1). Gynecologic examination under general anesthesia (all patients), cystoscopy, proctoscopy, and ultrasonography of the kidneys when involvement was suspected, were performed. Preoperative imaging methods were not standard for all; some had computerized tomography (CT), whereas some underwent magnetic resonance imaging (MRI). None of the patients had positron emission tomography-CT (PET-CT) data.

Lymph node dissection was performed via laparotomic extraperitoneal or laparoscopic extraperitoneal approach. Laparotomy (LPT) was performed through a left paramedian incision. The retroperitoneum was exposed by rolling the peritoneum medially till the psoas muscle and iliac vessels, bifurcation of the aorta, ovarian vessels and ureters were visualized. Paraaortic lymph nodes from the level of the common iliac bifurcation up to the level of the left renal vein were resected. Grossly enlarged (>2 cm) pelvic lymph nodes were also removed to improve the effect of radiotherapy. Laparoscopy (L/S) was performed using the technique described by Querleu et al. (6). Lymph nodes were defined macroscopically metastatic if they were palpable or of visible dimensions during the operation and positive in the pathology reports. In cases in which paraaortic lymph node metastasis was seen in frozen sections, scalene lymph node dissection was additionally performed.

In cases without paraaortic metastasis, external pelvic radiotherapy (5040 cGy) with intracavitary doses of 2800 cGy was performed. If the paraaortic metastasis was positive, 4500 cGy extended field radiotherapy was applied to the level of T_12_-L_1_. Concomitant chemotherapy was added to radiotherapy after the 2000s, which consisted of weekly cisplatin regimens (40 mg/m^2^ of body surface area, 25 mg/m^2^ in patients receiving paraaortic radiotherapy) intravenously with amifostine in some cases. Patients with scalene node metastasis were treated with chemotherapy plus palliative radiotherapy.

Patients were followed up every 3 months for 2 years, every 6 months until the fifth year following treatment, and yearly thereafter. In every follow-up, pelvic examination, abdominal ultrasonography, complete blood count, and blood chemistry were performed. Chest X-ray was performed yearly or in case of clinical suspicion. Thoracic and/or abdominal CT was requested when needed. The period from surgery to death or last visit was defined as OS. Follow-up time was evaluated as the time between surgery and the time of the patient’s last examination (death or last visit).

### Statistical analysis

The analysis of data was performed using SPSS for Windows, version 11.5 (SPSS Inc.; Chicago, IL, United States). OS and lifetime span were calculated according to Kaplan-Meier analyses and the log-rank test was used to determine factors that affected survival. Three- and 5-year survival rates, and expected lifetimes with 95% confidence intervals (CI) were calculated for each variable. Prognostic numeric and ordinal variables associated with survival were determined using the univariate Cox’s proportional hazards model. Hazards ratio with 95% CI and Wald statistics for each variable were calculated. Multivariate Cox’s regression analysis was used for the analysis of effects of risk factors that were found to affect survival. Variables with p values less than 0.25 were included in the multivariate analysis as risk factors. Statistical significance was considered at p<.05.

## RESULTS

When patients with follow-up less than 3 months (n=7) were excluded, the data of 233 patients were evaluated. The median age of the patients at the time of diagnosis was 52 years (range, 26-88 years). The median follow-up time was 28.4 months (range, 3-141 months). According to the FIGO clinical staging system, 183 (78.5%) patients were stage IIb, 8 (3.4%) were stage IIIa, 38 (16.3%) were stage IIIb, and 4 (1.7%) patients were stage IVa. The most common tumor type was squamous cell carcinoma (88.4%), followed by adenocarcinoma (6%) and adenosquamous carcinoma (1.7%). Tumor grade was valid in 82 patients and 72% of the tumors were grade 2. The cervical lesion was >4 cm in 142 (60.9%) patients and ≤4 cm in 91 (39.1%) patients. Extraperitoneal lymph node dissection was performed via L/S in 31(13.3%) patients and LPT in 202 (86.7%). Eight (25.8%) of the 31 patients who had L/S underwent LPT because of pneumoperitoneum failure.

The median paraaortic node yield was similar for both LPT and L/S groups [LPT group: 10 (2-33); L/S group: 13.5 (1-27), p=0.409]. Sixty-two of the 233 patients (27%) had paraaortic lymph node metastases. Metastases were microscopic in 36 patients, macroscopic but <2 cm in 20, and ≥2 cm in 6 patients. According to the operation notes, 57 (28%; n=57/202) patients in the LPT group had palpable paraaortic nodes. Twenty-nine (51%) of the patients with palpable paraaortic lymph nodes had paraaortic metastasis. The relation between palpable lymph nodes and metastasis was statistically significant (p<.001). Scalene lymph node dissection was performed in 55 patients, 9 (3.9%; n=9/55) of whom had metastases.

Major vascular injury [inferior vena cava (n=5), renal vein (n=2), inferior mesenteric artery (n=1)] occurred in 8 patients intraoperatively; all but one during LPT. Subcutaneous emphysema was seen in one patient during L/S. Postoperative complications were observed in 6.9% (n=16/233) of patients, all of which occurred after the LPT. The most common complication was wound disruption (n=10/16), followed by wound infection (n=2/16). The other complications were evisceration, deep vein thrombosis, hematoma formation in the wound, and subcutaneous fluid collection, one for each patient.

Preoperative MRI and CT of the lower abdomen were performed in 95 (41%) and 43 (18%) patients, respectively. MRI revealed pathologic-appearing lymph nodes in 8 patients who had metastatic nodes (n=8/20, sensitivity 40%), whereas none had pathologic findings in CT.

**Figure 1:**
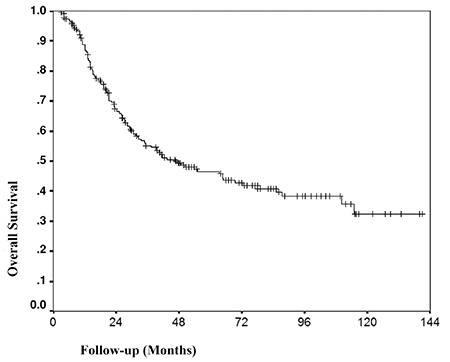
Kaplan-Meier analysis of cumulative survival rate of all patients

One hundred seventeen (50%) patients died in the follow-up period. The 3-year OS rate was 55.1% and the 5-year OS rate was 46.5%. Kaplan-Meier analysis of the survival of all patients is shown in Figure 1. Univariate analyses of categorical variables associated with OS are listed in Table 1.

**Figure 2:**
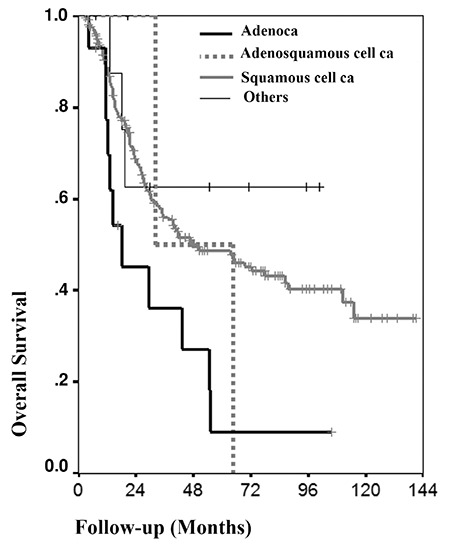
Kaplan-Meier analysis of cumulative survival rate according to histologic types

**Figure 3:**
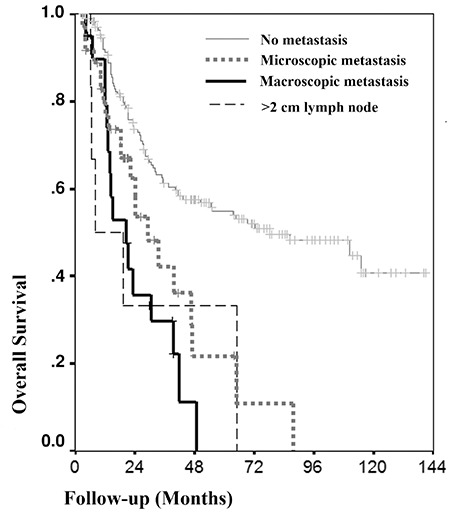
Kaplan-Meier analysis of cumulative survival rate according to paraaortic lymph node status

In the univariate analysis, stage of disease, number of metastatic paraaortic lymph nodes, tumor type, and paraaortic lymph node status were associated with OS (p<.001, p<.001, p=.039, and p<.001) (Table 1 and 2). OS worsened as the stage and number of metastatic paraaortic lymph nodes increased. Regarding the tumor type, the presence of adenocarcinoma affected OS negatively when compared with squamous cell carcinoma (Figure 2). The OS of patients with paraaortic lymph node metastases were significantly lower than that of patients without metastases (Figure 3). Scalene lymph node metastasis was not found associated with OS in the univariate analyses (p=.712).

In the multivariate Cox regression analysis, tumor type, stage, and presence of microscopic and macroscopic paraaortic metastases were independent prognostic factors of OS (Table 3). Prognosis worsened in the presence of adenocarcinoma, microscopic and macroscopic metastases, and advanced stages. The most important independent prognostic factor of survival was the presence of a macroscopic metastatic lymph node (Table 3).

## DISCUSSION

Based on the finding of better prognosis in patients who underwent surgical exclusion of paraaortic lymph node involvement compared with radiologically determined lymph node involvement, the importance of lymph node status affecting prognosis in locally advanced cervical cancer is emphasized in the current staging system of cervical cancer (7). Paraaortic metastasis (accepted as distant metastases, M1) upgrades the stage to IVb (FIGO staging), which previously did not exist in the clinical staging system (1). The NCCN 2015 guidelines recommend using surgical staging of patients with cervical cancer because the FIGO staging system does not include regional nodal metastasis and lymphovascular space invasion, which alters the treatment choice and success in both early-stage and advanced-stage disease (5). The amount of the radiation therapy is very critical depending on the paraaortic lymph node involvement. Concurrent chemoradiation using cisplatin-based chemotherapy is the most recent alternative, which is believed to increase survival rates (30-50% decrease in the risk of death compared with radiotherapy alone) (5).

Although there are many reports arguing the definite diagnostic procedure for nodal metastases, none of the imaging methods have been found superior to surgery (8). In addition to the limitation of detecting microscopic metastases, imaging methods are problematic regarding cost in low-income countries. Even PET-CT has not been considered satisfying for detecting real metastatic lymph nodes (sensitivity 36%) (9). We detected only 40% of the metastatic nodes using MRI and none with CT, but the low number of patients who underwent these imaging studies (95 patients had MRI, 43 patients had CT) may have decreased the sensitivity value.

Among the surgical techniques, which are more accurate way of detecting paraaortic metastases, transperitoneal or extraperitoneal lymph node dissection methods have been defined. The extraperitoneal approach was shown as superior to the transperitoneal technique with decreased bowel complications following radiotherapy (10). The extraperitoneal route, when performed laparoscopically, had additional advantages in reducing surgery- related problems and minimizing the time before radiotherapy (11). Wound complications were a problem in the LPT group in our study.

Laparoscopic extraperitoneal lymph node dissection has been performed since 1997 (12). In our series, only 31 patients were staged laparoscopically and OS did not differ compared with the group that underwent LPT. The number of removed paraaortic lymph nodes did not differ in either technique.

In our study, both intraoperative and postoperative complication rates were similar in LPT and L/S. The intraoperative complication rate in L/S was 6.5% in this study, which is close to the 5.7% rate reported in Querleu et al.’s study (13). Also, for the left paramedian incision group, intraoperative complications were observed in 3.4% patients, which may be acceptable. Postoperative complications consisted mostly of wound- related problems, which were not observed after L/S, favoring the laparoscopic technique.

Paraaortic involvement rates in the literature vary from 20% to 50% in locally advanced cervical cancer (14, 15). We found 27% paraaortic node involvement, which is comparable with reported results. Although clinical staging does not include lymph node metastasis, recurrence rates increased from 29% to 56% when metastases were detected (16). Also, survival rates vary greatly for the same stage when nodal involvement exists. Five-year survival was reported as 20-25% in patients with microscopic paraaortic lymph node metastasis in a study by Heaps and Berek (17), which is very close to our 5-year survival rates in the microscopic metastatic group. The authors argued for the benefits of extended field radiotherapy with chemotherapy in these patients who have no chance of survival without treatment. Sonoda et al. (18) reported a mean survival of 38.6 months in patients without paraaortic metastases and 26.5 months for metastatic patients with bulky tumors, but the follow-up period was much shorter. The five-year OS in our study was 15.5% and 54.9%, respectively, in the groups with and without paraaortic metastases.

Another important issue thought to be associated with survival is the extent of metastases. We found that OS did not differ significantly when the paraaortic nodal involvement was macroscopic or microscopic. Interestingly, Leblanc et al. (16) reported that OS was similar in patients who were node-negative and patients with microscopic nodal disease who received extended field (chemo) radiotherapy. This was not the only study showing poor prognosis in patients with macroscopic paraaortic metastases compared with microscopic metastases (14, 19, 20). Some authors reported the advantages of removing metastatic nodes (7, 15), but with the current findings, debulking may not be definitively associated with survival.

In the univariate analysis, stage of disease, number of metastatic paraaortic lymph nodes, tumor type, and paraaortic lymph node status were found associated with OS. Leblanc et al. (16) determined that tumor size greater than 5 cm was associated with poor prognosis, but we did not find tumor size as a prognostic factor. In the multivariate Cox regression analysis, tumor type, stage, presence of microscopic and macroscopic paraaortic metastases were independent prognostic factors of OS. Regarding tumor type, there are conflicting data in the literature. Turan et al. (21) reported that tumor type, grade, tumor size, and parametrial invasion did not affect survival except lymphovascular space invasion in stage Ib cervical cancer. Unlike previous studies that reported no significant prognostic difference between squamous cell carcinoma and adenocarcinoma type (22, 23), we found that adenocarcinoma of the cervix worsened the prognosis and decreased survival of patients.

There are few studies about scalene nodal metastases in cervical cancer. In the present study, no survival difference was detected between patients with positive and negative metastatic scalene lymph nodes who had scalene lymph node dissection. Scalene node involvement means disseminated disease necessitating palliative treatment, but the current results showed that the actual prognostic factor was paraaortic nodal status. Supporting this, both paraaortic metastases and scalene metastases are considered as stage IVb in the surgical staging system (5).

One of the limitations of this study was the retrospective design. Detailed radiotherapy data and post-radiotherapy complication rates were missing because subsequent treatment of some patients including radiotherapy had been completed in different centers.

Despite the limitations, this retrospective study has a good number of patients. Extraperitoneal lymph node dissection in patients with locally advanced cervical cancer has been investigated for years and has been shown to have a significant prognostic effect (7). There is an ongoing phase III trial aiming to determine as to whether laparoscopic surgical staging improves survival, which we believe will guide management choices in the future (24). Once more, we showed that paraaortic lymph node metastasis was the most important prognostic factor affecting the survival of the patients. The most striking result is that surgery gives clues about the prognosis of the patient better than the clinical stage. Surgical staging aids in planning adjuvant therapy, prevents unjustifiable extended field radiotherapy and further complications, and thus decreases morbidity. Treatment plans of patients with locally advanced cervical cancer should be performed according to the results of lymphadenectomy, which is applicable for every patient. Extraperitoneal lymphadenectomy might contribute to improvement of survival.

## Figures and Tables

**Table 1:**
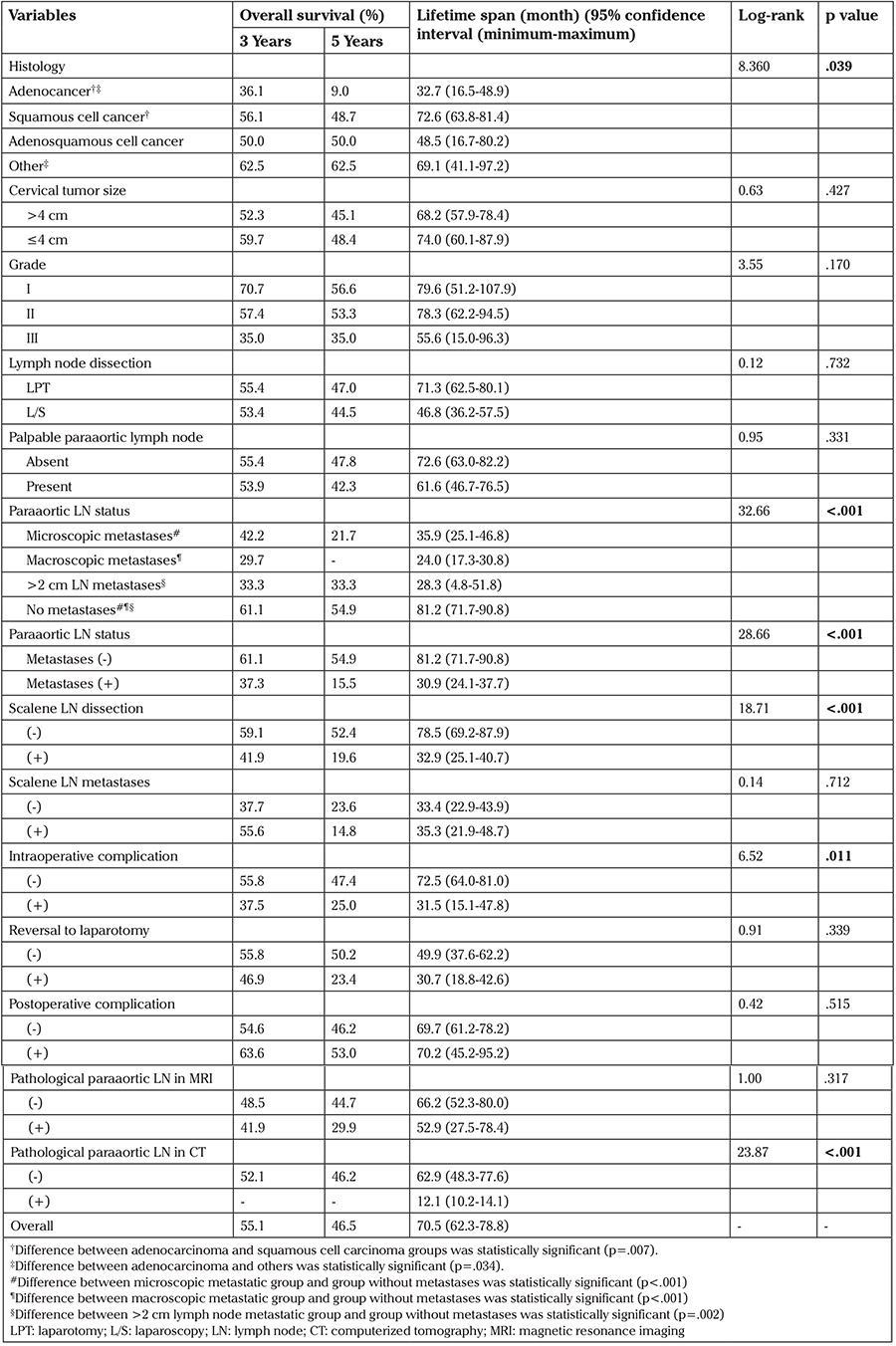
Univariate Kaplan-Meier survival analysis results of categorical variables that may affect overall survival

**Table 2:**
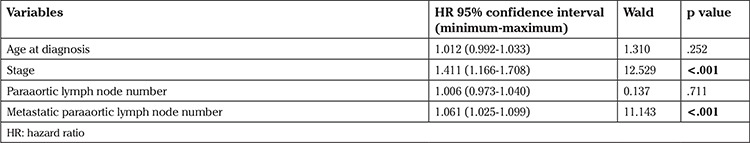
Results of univariate Cox regression analyses of numerical and ordinal variables that may be associated with survival

**Table 3:**
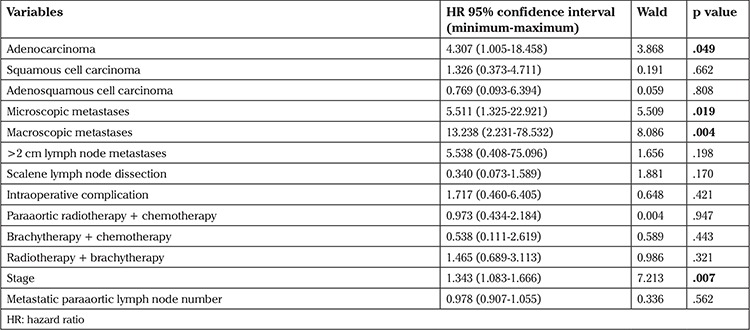
Evaluation of all factors that may be associated with overall survival with multivariate Cox hazards regression analyses
